# Domestic Correspondence

**Published:** 1891-06

**Authors:** 


### Domestic Correspondence.

To the Editor:

At the annual meeting of the Chicago Dental Society, held Tuesday evening, April 7, 1891, the following officers were elected for the ensuing year:

D. M. Cattell, President; J. W. Wassall, First Vice-President; E. M. S. Fernandez, Second Vice-President; L. L. Davis, Recording Secretary; T. L. Gilmer, Corresponding Secretary; E. D. Swain, Treasurer; A. W. Harlan, Librarian.

*Executive Committee.*—J. A. Dunn, G. H. Cushing, E. Noyes.

*Board of Censors.*—B. S. Palmer, G. J. Dennis, R. M. C. Paine.

Thomas L. Gilmer,
*Corresponding Secretary.*

April 9, 1891.

---

To the Editor:

The commencement exercises of the University Dental College, Department of the Northwestern University of Chicago, were held in connection with the Medical Department, Chicago Medical College, at Central Music Hall, on April 28, at 2.30 p.m.

The degrees were conferred upon the graduating classes by President Henry Wade Rogers, LL.D., and the doctorate address was delivered by Professor N. S. Davis, Sr., dean of the Medical Faculty.

The following is the list of the dental graduates:

Ellsworth Goldthorp, Iowa; William Edward Harper, Illinois; Alexander Clarence Murchison, Illinois; William B. Winget, Illinois.

John S. Marshall,

*Dean.*

Chicago, Ill., April 30, 1891.

